# Investigation of extramammary sources of Group B *Streptococcus* reveals its unusual ecology and epidemiology in camels

**DOI:** 10.1371/journal.pone.0252973

**Published:** 2021-12-03

**Authors:** Dinah Seligsohn, Chiara Crestani, Nduhiu Gitahi, Emelie Lejon Flodin, Erika Chenais, Ruth N. Zadoks

**Affiliations:** 1 Department of Animal Health and Antimicrobial Strategies, National Veterinary Institute, Uppsala, Sweden; 2 Department of Clinical Sciences, Swedish University of Agricultural Sciences, Uppsala, Sweden; 3 Institute of Biodiversity, Animal Health and Comparative Medicine, University of Glasgow, Glasgow, United Kingdom; 4 Department of Public Health, Pharmacology and Toxicology, University of Nairobi, Nairobi, Kenya; 5 Uppsala Small Animal Clinic, Uppsala, Sweden; 6 Department of Disease Control and Epidemiology, National Veterinary Institute, Uppsala, Sweden; 7 Sydney School of Veterinary Science, Faculty of Science, The University of Sydney, Camden, NSW, Australia; INRA, FRANCE

## Abstract

Camels are vital to food production in the drylands of the Horn of Africa, with milk as their main contribution to food security. A major constraint to camel milk production is mastitis, inflammation of the mammary gland. The condition negatively impacts milk yield and quality as well as household income. A leading cause of mastitis in dairy camels is *Streptococcus agalactiae*, or group B *Streptococcus* (GBS), which is also a commensal and pathogen of humans and cattle. It has been suggested that extramammary reservoirs for this pathogen may contribute to the occurrence of mastitis in camels. We explored the molecular epidemiology of GBS in camels using a cross-sectional study design for sample collection and phenotypic, genomic and phylogenetic analysis of isolates. Among 88 adult camels and 93 calves from six herds in Laikipia County, Kenya, GBS was detected in 20% of 50 milk samples, 25% of 152 nasal swabs, 8% of 90 oral swabs and 3% of 90 rectal swabs, but not in vaginal swabs. Per camel herd, two to four sequence types (ST) were identified using Multi Locus Sequence Typing (MLST). More than half of the isolates belonged to ST617 or its single-locus variant, ST1652, with these STs found across all sample types. Capsular serotype VI was detected in 30 of 58 isolates. In three herds, identical STs were detected in milk and swab samples, suggesting that extramammary sources of GBS may contribute to the maintenance and spread of GBS within camel herds. This needs to be considered when developing prevention and control strategies for GBS mastitis. The high nasal carriage rate, low recto-vaginal carriage rate, and high prevalence of serotype VI for GBS in camels are in stark contrast to the distribution of GBS in humans and in cattle and reveal hitherto unknown ecological and molecular features of this bacterial species.

## Introduction

In the arid and semi-arid lands of the Horn of Africa, nomadic pastoralism is common, and livestock is mainly kept for sustenance [1]. Camels are well-adapted to surviving under arid conditions despite limited access to water and feed [2] and have long been kept for milk and meat by pastoralist communities in this region [1]. The importance of camels is likely to grow because of climate change, resulting in prolonged and recurrent droughts and erratic rainfall [3]. Because of this, camel keeping is increasing in traditional cattle-keeping communities in Kenya [4]. Furthermore, the demand for camel milk has grown over the past decades. Consequently, the camel milk sector in Kenya is currently undergoing substantial changes despite being hampered by lack of resources and adequate infrastructure [5].

Mastitis, inflammation of the udder most often caused by bacterial infection, is a common constraint to milk production in dairy camel herds in this region [6, 7]. This has implications for household income and food security, as well as animal and public health [8]. *Streptococcus agalactiae*, also referred to as Group B *Streptococcus* (GBS), is one of the most important udder pathogens in camels, often resulting in chronic infections [7, 9]. In dairy cattle, GBS was considered an obligate intramammary pathogen, but this paradigm has recently been challenged as GBS has been found in the environment and body sites other than the mammary gland (e.g., in faeces and in swabs taken from the rectum, vagina and throat) of cattle in South America, North America and Europe [10–12]. Similarly, in camels, GBS has been isolated from the nasal and vaginal mucosa of clinically healthy camels [13], exemplifying extramammary sources of GBS that may constitute a reservoir for intramammary infections. Fischer and colleagues [14] mapped specific sequence types (STs) and capsular types of camel GBS to various clinical presentations and body sites, but the role of healthy carriers in maintaining a GBS population, and their involvement in GBS mastitis epidemiology have not been investigated.

In this study we used phenotypic, genomic and phylogenetic methods to investigate GBS diversity in isolates from camel milk and extramammary sources, with a specific focus on the potential role of extramammary strains in mastitis, and the overall objective to expand the knowledge basis for mastitis control in camels.

## Materials and methods

### Ethics statement

The study was approved by the National Commission for Science, Technology and Innovation, Nairobi, Kenya (Permit number: NACOSTI/P/19/84995/13088). Prior to sampling, animal owners and herders received oral or written information about the purpose of the study and the sampling procedures, and consent to participate was obtained. Participants were informed that inclusion in the study would be anonymous and that they could withdraw from the study at any time. No additional permissions for sample collection and analysis were required.

### Study area and selection of herds

The study was conducted in Laikipia County, Kenya. Laikipia is classified as semi-arid with an average annual rainfall of 639 mm. The land is predominantly used for ranching, including large-scale ranches with hired labour, and for pastoralist animal production in the north of the county. Smallholder farmers with subsistence agriculture dominate in the south of the county [15]. The total camel population measures 9,800 [16] and acquisition of camels among pastoralist groups without a tradition of camel keeping is increasing [17]. Most camels are semi-sedentary in this region. Camel calves are kept with their mothers on pasture during the day and separated from their dams during the night, when animals are kept in “bomas” (animal enclosures traditionally made from twigs and branches or other more solid materials). The study area was selected because of its considerable and growing camel population, and the presence of a camel milk collection center. Sampling took place during the wet season, November 2019. Herds (n = 6, A-F) were selected on the basis of subclinical mastitis prevalence data [18].

#### Sampling and bacteriological culture

Sampling was carried out during the early morning milking between 4 am and 8 am. Calves were sampled before being released from their overnight boma to suckle their mother to initiate milk let down. All lactating camels were screened using the California Mastitis Test [19] and scored according to the Nordic scale (1–5) [20], where 1 signifies no change in viscosity and 5 signifies a marked increase in viscosity with formation of a distinct gel peak. Viscosity is an indicator of the severity of inflammation, which in turn is an indicator for the likelihood of intramammary infection. From camels with a CMT-score of 2 or higher, composite milk samples were collected aseptically in sterile plastic 50 mL vials to detect bacteria causing intramammary infection. Milk samples were kept cold during transport using cooler bags and frozen at -20°C within 4 hours from the time of collection. Swab samples were collected from all lactating camels and their calves using sterile flocked nylon swabs (e-swab, COPAN diagnostics Inc. Murrieta, CA, USA). Prior to swabbing, a clinical assessment of each sampling site was carried out. Clinical abnormalities, such as nasal or vaginal discharge, swelling, lesions or diarrhoea were noted. Swabs were taken from the nasal and vaginal mucosa of lactating camels, and from the nasal, oral and rectal mucosa of their respective suckling calves. The nasal mucosa was sampled by opening the nostrils and swabbing the nasal cavity on both sides with the same swab. The vaginal mucosa was sampled by separating the labia, inserting the swab and rolling it against the vaginal wall. Oral samples were collected by inserting the swab into the oral cavity of the calf and rolling it against the buccal and pharyngeal mucosa. The rectal mucosa was sampled by inserting the swab in the rectum and gently rolling it against the rectal wall. Swab samples were kept refrigerated at 4-8°C and processed at the Department of Public Health, Pharmacology and Toxicology, University of Nairobi, Nairobi, Kenya, within 1 to 10 days from collection.

For processing, milk samples were thawed at room temperature. Ten μL of milk were cultured on modified Edward’s agar (EA) (Oxoid, CM0027), and incubated aerobically for 24 and 48 hours at 37°C before final examination. Milk samples that were negative for GBS on direct culture were enriched in Todd Hewitt (TH) broth (Oxoid, CM0189) at 37°C for 18 to 24 hours and re-cultured on EA. Catalase-negative, KOH-negative colonies, displaying blue pigmentation, with or without β-haemolysis, were subcultured on blood agar (Oxoid, CM0271) containing 5% defibrinated sheep blood.

Swab samples were enriched in TH broth at 37°C for 18–24 hours prior to plating. A calibrated loop (10 μL) was used to plate the enrichment on EA and the plates were incubated aerobically at 37°C for 18–48 hours. Colonies that showed the same phenotypic and biochemical characteristics as described above were subcultured on EA and incubated at 37°C for 18–48 hours. All CAMP (Christie-Atkins-Munch-Peterson)-positive [21] bacterial isolates (n = 58) were confirmed as GBS using a slide latex agglutination test (Streptex Latex Agglutination Test, ThermoFisher Scientific Inc., Waltham, MA, USA). GBS isolates were subcultured on blood agar to assess purity and inoculated on stab agars (SVA, Uppsala, Sweden) that were incubated aerobically for 8–12 hours in 37°C before storage at 4–8°C. Stab agars were transported at ambient temperature to the National Veterinary Institute (SVA), Uppsala, Sweden, which took 24 hours, and then recultured on blood agar containing 5% defibrinated horse blood (SVA, Uppsala, Sweden) and incubated aerobically for 24–48 hours at 37°C. Species identity was confirmed using matrix assisted laser desorption ionization-time of flight mass spectrometry analysis (MALDI-ToF MS) [22]. The bacteria were analyzed in duplicates. Criteria for species identification were as follows: a score of ≥2 indicated identification at species level, 1.80 to 1.99 at genus level, and <1.80 no identification. Species identification was performed using a custom-made database including the Bruker databases no. 5627 and no. 5989.

#### DNA extraction and sequencing

Extraction of DNA was carried out from all confirmed GBS isolates (n = 58), using a magnetic bead-based method. A calibrated loop (1 μL) was used to suspend colony material in 600 μL nuclease free water (Sigma-Aldrich, St Louis, MO, USA) and mixed with 0.1 mm silica beads (BioSpec Products Inc., Bartlesville, USA). The suspension was added to the FastPrep24 (MP Biomedicals LLC, Irvine, CA, USA) and run at 6.5 m/s for three 2-minute cycles. DNA was extracted from 200 μL samples using the IndiMag Pathogen kit (Indical Bioscience GmbH, Leipzig, Germany) and eluted in nuclease free water. The Invitrogen Qubit 3.0 Fluorometer and the Qubit dsDNA BR Protein Assay kit (ThermoFisher Scientific Inc., Waltham, MA, USA) were used to measure DNA concentrations, which were adjusted to 7.5 ng/μL. Library preparation and whole genome sequencing were performed by Clinical Genomics, Science for Life Laboratory (Clinical Genomics, Solna, Sweden) on the Illumina NovaSeq (Illumina, Inc. CA, US), resulting in paired-end libraries of 150 bp read length.

#### Antimicrobial susceptibility testing

GBS isolates were tested for presence of phenotypic antimicrobial resistance by determination of minimum inhibitory concentration (MIC). Testing (but not interpretation) was performed according to standards of the Clinical and Laboratory Standards Institute [23] using a broth microdilution method, cation-adjusted Mueller-Hinton broth, Sensititre^TM^ STAFSTR panels (TREK Diagnostic System, UK) and Sensititre^TM^ NLD1GNS panels (TREK Diagnostic System, UK). As quality control, a strain of *Staphylococcus aureus* ATCC 15019 was tested in parallel with the isolates; results were within acceptable ranges. The MIC were determined for the following compounds: cephalotin, clindamycin, enrofloxacin, erythromycin, gentamicin, nitrofurantoin, penicillin, tetracycline and trimethoprim-sulfamethoxazole. GBS specific epidemiological cut-off (ECOFF) values issued by the European Committee on Antimicrobial Susceptibility Testing (EUCAST; clindamycin, erythromycin, nitrofurantoin, penicillin and tetracycline) were used to classify isolates as wild type (WT; the ECOFF based counterpart of susceptibility, which is defined based on clinical cut-off values) or non-wild type (non-WT, the ECOFF-based counterpart of resistance), with clinical breakpoints from SVA used for the remaining compounds (cephalotin and trimethoprim-sulfamethoxazole). *Streptococcus* species have a low inherent susceptibility to quinolones and an ECOFF value is not defined by EUCAST. MIC for enrofloxacin was determined in this study but not classified as WT, non-WT, susceptible or resistant due to the lack of available cut-off values. Seventeen isolates showed growth at the highest concentration of gentamicin provided at 8 μg/mL and were subsequently retested on the Sensititre^TM^ NLD1GNS panel with a wider range for gentamicin (up to 32 μg/mL). SRST2 was used to detect antimicrobial resistance (AMR) genes from raw reads with the ARG-ANNOT v3 database [24].

#### Lactose typing

Lactose fermentation was assessed phenotypically by inoculating each of the isolates onto bromocresol purple lactose agar (SVA, Uppsala, Sweden). Lactose fermentation was indicated by a yellow colour change, whereas the absence of colour change was interpreted as negative for lactose fermentation. *Escherichia coli* ATCC35218 and *Proteus mirabilis* CCUG26767 were used as positive and negative controls, respectively. Plates were incubated aerobically at 37°C and checked for colour change at 24, 48, and 72 hours and after one week of incubation. The presence of the lactose operon (Lac.2) [25] was investigated with a BLASTn v2.9.0 search [26] based on a database of five known Lac.2 genotypic variants [27, 28]. Thresholds for positivity were set at 90% for both sequence identity (ID) and query coverage (QC). Lac.2-negative genomes were confirmed by manual scanning of annotation files obtained using Prokka v1.14.6 [29], searching for genes associated with the metabolism of lactose. To further support these findings, we conducted a PCR targeting a ≈2.5-kbp region straddling *lacEG*. Positive and negative controls were selected from among the study isolates based on genomic detection of Lac.2.

#### Phylogenetic and statistical analysis

Reads were filtered and trimmed with ConDeTri suite v2.3 [30]. *De novo* assembly was performed using SPAdes v3.13.1 [31] and assembly quality was checked with QUAST v5.0.2 [32]. Bacterial species identity was confirmed with KmerFinder v3.2 [33]. All assembled genomes passed quality control. Multi locus sequence typing (MLST) was carried out with SRST2 v0.2.0 [34] and capsular serotype was detected *in silico* using a standard method [35]. All sequencing data from this study are available on NCBI under the Bioproject number PRJEB44246. Isolate metadata and individual accession numbers (ERR, ERS and ERX numbers) can be found in the (S1 Dataset).

Snippy v4.6.0 (https://github.com/tseemann/snippy) was used for alignment of the core genome. ILRI112, which is an ST617 genome of a GBS isolate collected from an abscess in a camel from Kenya, was used as reference (GenBank Accession number HF952106) [36]. A maximum likelihood tree was inferred using RAxML-NG v0.9.0 [37] under a GTR+G model. A map of herd coordinates was created with ggmap with R, in RStudio (v4.0). The shapefiles for county borders and roads were downloaded from ArcGIS (https://www.esri.com/en-us/arcgis/products/arcgis-online/overview), these files are open to access, use and share, under the ArcGIS terms of use. All figures were edited using Inkscape (www.inkscape.org).

Data editing was carried out in Excel (Microsoft Corp., Redmond, WA, USA). Associations between categorical variables were tested for statistical significance using Pearson’s chi-square test or Fisher’s exact test when suitable. A *p-*value of 0.05 or lower was considered significant. All statistical analyses were performed in STATA (Stata Statistical Software, release 13.1; StataCorp LP, College Station, TX).

## Results

In all, 50 milk samples, 88 nasal mucosa swabs and 88 vaginal mucosa swabs were collected from 88 adult camels in six herds (A to F). Swab samples (n = 244) were also collected from the nasal (n = 64), oral (n = 90) and rectal (n = 90) mucosa of 93 suckling calves. Due to occasional difficulties in restricting camels for sampling or camels being released on pasture before the sampling was completed, some sample categories are missing for a few individuals in herds A, D, E and F.

### Herd demographics

**Fig 1:**
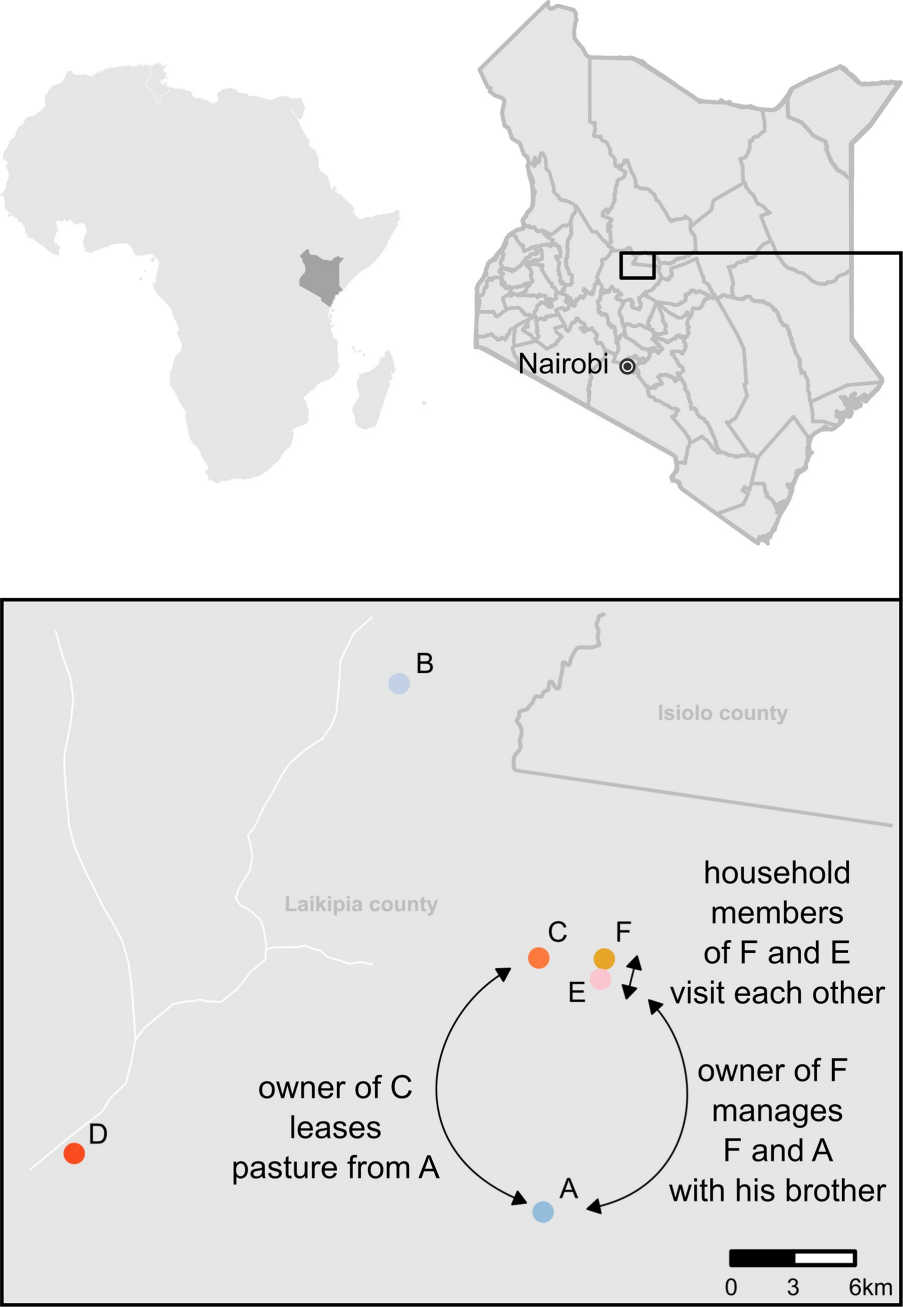
Geographical location of six dairy camel herds (A-F) in Laikipia County, Kenya. Arrows indicate known epidemiological links between herds. The map of herd coordinates was created with ggmap with RStudio in R (v4.0).

Herds belonged to different management systems, but all camels were herded during the day and kept overnight in bomas (**Table 1**) with lactating dams separated from their calves. Three of the herds (A, B and D) were kept under ranch management, i.e., herds belonged to private landowners with no tradition of keeping camels who employed herdsmen, most of them from pastoralist communities and skilled in camel management, to handle their livestock. One herd (C) was semi-commercial, owned by pastoralists, kept under traditional pastoralist conditions but with employed herders. Two herds (E-F) were managed by Maa-speaking families that came from a cattle-keeping tradition but had added camels to their herds during the last two decades. They kept their camels at the homestead together with many other animal species, and milk was mainly used for household consumption. Management characteristics are shown in **Table 1.** Estimated average herd size was 87 and the average number of lactating camels seventeen. Camels were mainly of Somali breed (n = 62) or Turkana breed (n = 15), followed by crossbreeds (n = 6) and other breeds (n = 4), information on breed was missing for one camel. Herd location is shown in **Fig 1**.

**Table 1:** Herd demographics and management characteristics of six dairy camel herds from a study into carriage and shedding of group B *Streptococcus* conducted in November 2019 in Laikipia County, Kenya.

Herd and management characteristics	Herd					
	A	B	C	D	E	F
**Management system**	Ranch	Ranch	Pastoralist	Ranch	Homestead	Homestead
**Mobile/permanent boma**	Mobile	Mobile	Mobile	Permanent	Permanent	Permanent
**Boma material**	Metal fence	Acacia	Acacia	Wooden fence	Acacia	Acacia
**Total herd size** ^**1**^	76	75	180	86	53	53
**Number of lactating camels**	19	23	18	16	17	8
**Milking frequency (times/day)**	1	2	2	4	2	2
**Post-milking cleaning of hands** ^**2**^	Yes	Yes	No	No	No	No
**Milking order**	No	No	Newly calved camels milked first	Oldest camels milked first	Newly calved camels milked first	Newly calved camels milked first
**Selling milk**	Yes	Yes	Yes	Yes	Yes	No
**Watering interval (days)**	2	0	1	0	1	0
**Other animals kept in contact with camel herd**	Cattle, poultry, sheep	No	No	No	Cattle, poultry, sheep, goats, dogs	Cats, poultry, sheep, goats, dogs
**New camels added to the herd within the last year**	No	No	No	Yes	No	No

^1^For herds not belonging to ranches, herd size was regarded as confidential, and a rough estimate was given by the herders.

^2^No pre-milking washing of hands was carried out in any of the herds.

### Mastitis and GBS

Milk samples originated from animals with CMT ≥2 in at least one quarter, which was observed in 50 of 88 lactating camels (57%), and in each herd. Most of these mastitis cases were subclinical. Only nine camels displayed symptoms of acute mastitis, such as swollen udder, pain or deviations of the milk appearance (changes in colour, consistency, clotting or blood). Previous udder problems were reported for 25 of 88 camels and ten of 88 camels had at least one blind (non-milk producing) quarter. Four calves from three herds were observed to suckle from camels other than their mother (cross-suckling). GBS was isolated from milk of ten (20%) CMT-positive camels in four herds; seven of these camels had reportedly had long lasting udder problems, and there was a positive association between a previous history of mastitis and isolation of GBS from milk (*p* = 0.004).

### Extramammary sources of GBS

Extramammary GBS were detected in all six herds, in 24 of 88 nasal swabs from adult camels and in 19 of 95 calves, including 14, 7 and 3 nasal, oropharyngeal and rectal swabs, respectively (**Table 2**). Swabbed tissues and sites appeared clinically normal. The only exception was in herd C, where orf-like lesions were present on the muzzle of all calves. The most common site of GBS isolation was the nasal mucosa (38/152 samples). Only seven oral swabs from three herds and three rectal swabs from two herds were positive for GBS. No vaginal swabs were positive for GBS. In two herds (E and F), GBS were exclusively detected in extramammary samples.

**Table 2:** Group B *Streptococcus* (GBS) detected in milk and extramammary body sites of camels from six herds in Laikipia, Kenya, November 2019.

Herd	Adults			Calves			Total GBS isolates
	Milk samples	Vaginal samples	Nasal samples	Nasal samples	Oral Samples	Rectal samples	
**A**	2/12	0/22	5/22	n/a^1^	0/26	2/26	9
**B**	4/14	0/23	3/23	3/23	1/23	1/23	12
**C**	1/9	0/19	3/19	4/19	0/19	0/19	8
**D**	3/10	0/11	4/11	1/9	0/9	0/9	8
**E**	0/3	0/7	4/7	3/8	2/8	0/8	9
**F**	0/2	0/6	5/6	3/5	4/5	0/5	12
**Total**	10/50	0/88	24/88	14/64	7/90	3/90	58/470
**Prevalence**	20%	0%	27%	22%	8%	3%	12%

(GBS-positive samples/total number of samples, with prevalence expressed as % for column totals).

^**1**^No nasal swabs were collected from calves in herd A.

For nine camels, GBS were isolated from two sampling sites in the same individual (adults: n = 3; calves: n = 6).

### Diversity of sequence types and serotypes

**Fig 2:**
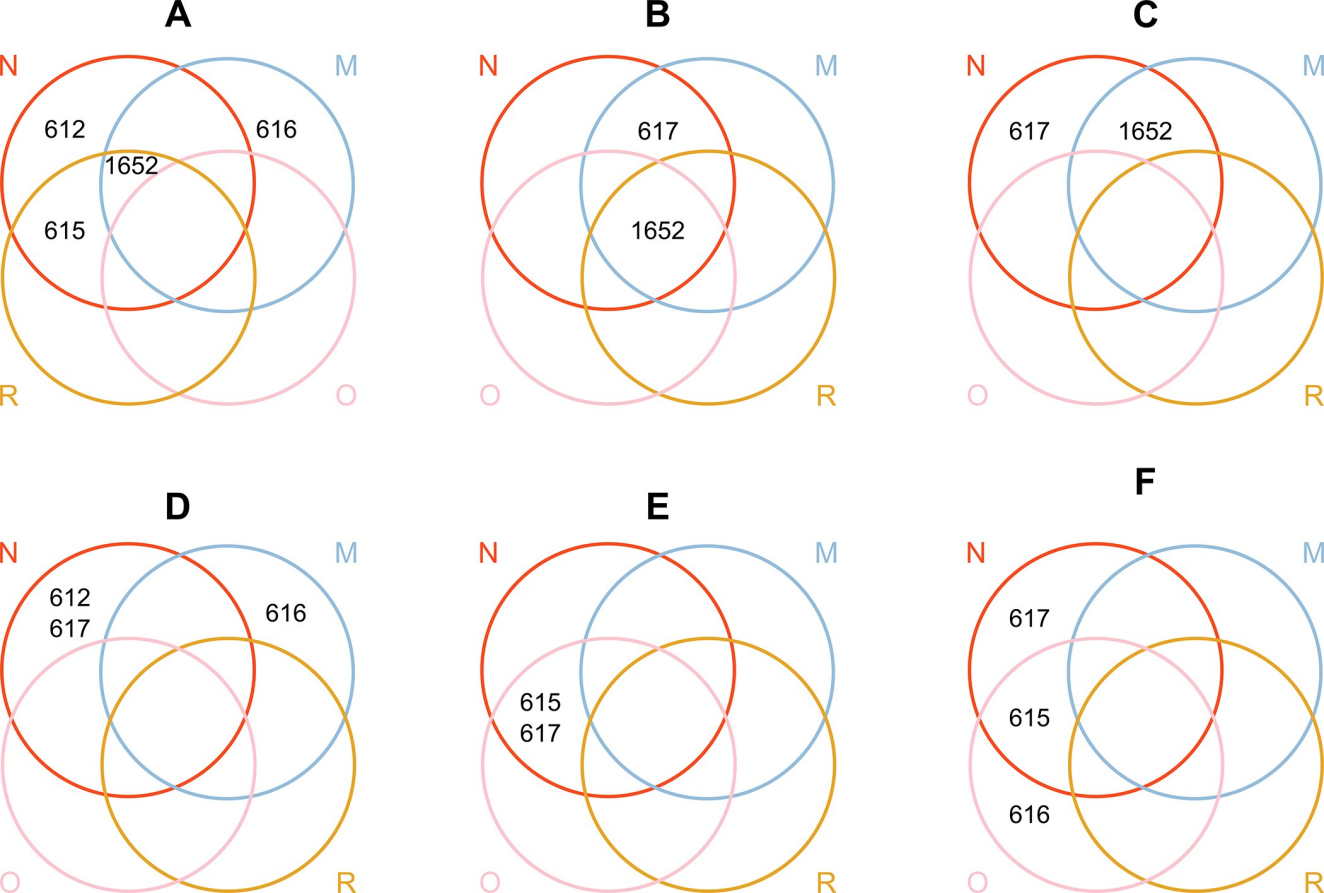
Visualisation of the herd distribution of group B *Streptococcus* sequence types (STs) isolated from different sampling sites in six camel herds (A-F) in Laikipia County, Kenya, November 2019. Colour of the rings and capital letters correspond to sampling sites; N = nasal swab (red), R = rectal swab (yellow), O = oral swab (pink), M = milk sample (blue). Note that the position of rings for herd A is modified to allow for visualisation of the data.

One DNA extract did not pass quality control for sequencing and was excluded from analysis. Five sequence types (ST) were identified, all belonging to previously described camel-associated clonal complexes. More than half of the isolates belonged to ST617 (n = 17) or its single locus variant (SLV) ST1652 (n = 19), followed by ST615 (n = 10), ST612 (n = 6) and ST616 (n = 5). Within-herd diversity varied between herds: in one herd (A), isolates belonged to four STs, in two herds (D and F), isolates belonged to three STs and in the remaining three herds (B, D and E), isolates belonged to two STs (**Fig 2**). Within-host diversity was found in six camels, in which isolates belonged to two STs. There was a significant association between ST and sampling site (*p* = 0.005). For example, four of five ST616 isolates were found in milk, and five of six ST612 isolates and 15 of 17 ST617 isolates were found in nasal swabs (**Table 3)**. ST1652 was isolated from all GBS-positive sample types whereas ST617 was found in nasal and oral swabs and in milk, ST612 in nasal and oral samples, and ST616 in milk samples and an oral sample (**Fig 2**.).

**Table 3:** Distribution of sequence types (STs) of Group B *Streptococcus* (GBS) isolated from camel milk and extramammary body sites collected from six herds in Laikipia County, Kenya, November 2019.

Sequence type	Milk samples		Nasal samples		Rectal samples		Oral samples	
	Isolates n	Herds	Isolates	Herds	Isolates	Herds	Isolates	Herds
**ST612**	0	0	5	3	0	0	1	1
**ST615**	0	0	6	2	1	1	3	1
**ST616**	4	2	0	0	0	0	1	1
**ST617**	1	1	15	5	0	0	1	1
**ST1652**	5	3	11	3	2	2	1	1
**Total isolates**	10		37		3		7	

**Fig 3:**
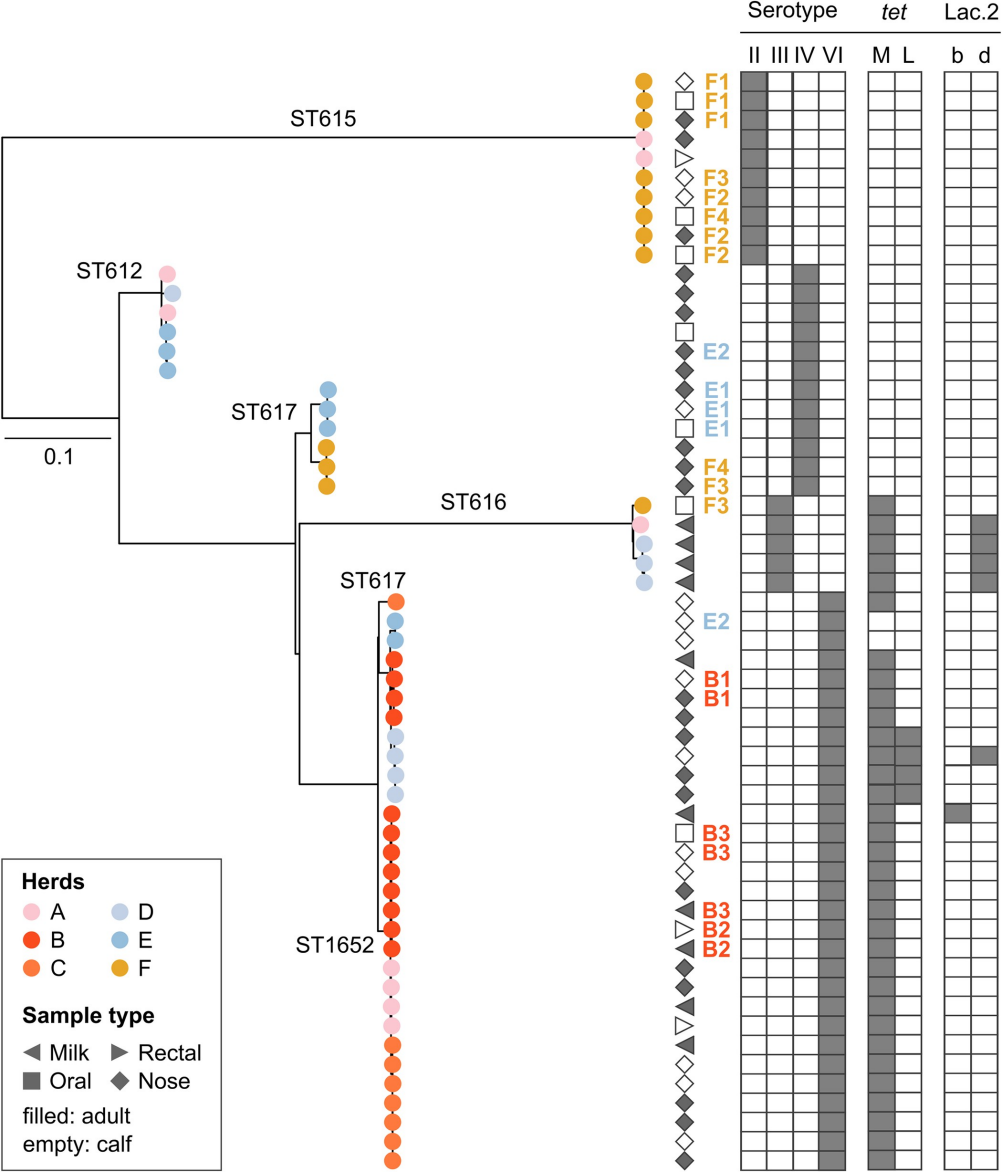
Maximum likelihood phylogenetic tree based on a core genome alignment for 57 group B *Streptococcus* isolates from milk and nasal, rectal and oral mucosa of dromedary camels in Kenya. Leaf colours indicate herd of origin with sequence types (STs) indicated on the branches. Geometric shapes correspond to sample source, while age category (adult or calf) is indicated by shading. Combinations of letters and numbers show adult-calf pairs within herds (B1, B2, B3, E1, E2, F1, F2, F3, F4) with colour corresponding to herd of origin. Grey blocks mark capsular serotype, tetracycline resistance genes, and the presence of a lactose operon in the genome (variants Lac.2b and Lac.2d). Tree was rooted at midpoint.

Four capsular serotypes were detected *in silico*. The most common serotype was serotype VI (n = 30), followed by serotype IV (n = 12), serotype II (n = 10) and serotype III (n = 5). Serotypes II and III were only found in one ST each, whereas serotypes IV and VI were found in two STs. Except for ST617, STs were associated with a single serotype (*p*<0.001) **(Table 4)**. Serotypes matched perfectly with phylogenetic groups (**Fig 3**), with no indication of capsular switching within lineages.

**Table 4:** Distribution of molecular serotypes and sequence types (STs) among 57 group B *Streptococcus* isolates collected from milk, nasal, rectal and oral mucosa from lactating camels and their calves in six herds in Laikipia County, Kenya, 2019.

Capsular serotype	ST612	ST615	ST616	ST617	ST1652	Total
II	-	10	-	-	-	10
III	-	-	5	-	-	5
IV	6	-	-	6	-	12
VI	-	-	-	11	19	30
**Total**	**6**	**10**	**5**	**17**	**19**	**57**

### Phylogenetic analysis

In the core genome phylogeny, five main lineages can be observed (**Fig 3**). Four lineages corresponded to a single ST (ST612, ST615, ST616 and ST617 respectively) and the fifth and largest lineage included ST617 and its SLV, ST1652. Two of these lineages involved isolates from only nasal and oral mucosa, while ST616 was primarily found in milk samples. To a large extent, isolates from the same herd clustered close to each other within each lineage. Nine adult-calf pairs were GBS-positive, with two or three GBS isolates per pair. For six adult-calf pairs, isolates were genetically related (B1, B2, B3, E1, F1, F2, **Fig 3**). Only two of these adult-calf pairs involved an isolate from the mother’s milk (B2 and B3). In one adult-calf pair (B2, **Fig 3**), the isolate from the milk clustered with an isolate from the rectal mucosa of the calf. In the other pair (B3), the isolate from the mother’s milk clustered with one isolate from the oral mucosa and one isolate from the nasal mucosa of the calf. The other four closely related adult-calf pairs consisted of nasal isolates from both the mother and calf. In the remaining three adult-calf pairs, isolates belonged to different lineages (E2, F3, F4, **Fig 3**).

### Antimicrobial susceptibility and lactose typing

The phenotypic susceptibility testing revealed non-WT phenotypes for tetracycline (MIC above 1 μg/mL) in 33 of 57 GBS isolates. MIC values for all other categories of antibiotics were within MIC ranges classified as WT or susceptible (for ECOFF or clinical breakpoints, respectively) with the exception of enrofloxacin, for which ECOFF and clinical breakpoint were lacking. The distribution of MIC values is shown in **Table 5.** MIC values for each isolate are presented in the **S1 Dataset**. The proportion of tetracycline non-WT isolates differed significantly between herds (*p*<0.001). In herd B and C, all isolates were classified as non-WT whereas in herd E, all isolates were WT. In all phenotypically tetracycline non-WT isolates, the *tet*(M) gene was found. In addition, four nasal isolates from one herd (herd D) harboured the *tet*(L) gene. No resistance genes were found in the phenotypically WT isolates. Isolates originating from milk were more likely to be tetracycline non-WT (10 out of 10) than isolates from extramammary sources (*p* = 0.015). There was an association between ST and tetracycline non-WT (*p*<0.001), with all isolates belonging to ST1652 (n = 19) and ST616 (n = 5) being resistant.

**Table 5:** Distribution (percentage of isolates) of minimum inhibitory concentration (MIC) and prevalence of non-WT or resistance (R, shown as percentage) for group B *Streptococcus* (n = 58) isolated from lactating camels and their calves in Laikipia County, Kenya, 2019^1^.

		Distribution (%) of MICs (μg/mL)											
Test agent	% R	≤0.03	0.06	0.125	0.25	0.5	1	2	4	8	16	32	64
Cephalotin	0						100						
Clindamycin	0					100							
Enrofloxacin	NA					70.7	29.3						
Erytromycin	0					100							
Gentamicin	0							5.2	65.5	29.3			
Nitrofurantoin	0										100		
Penicillin	0	27.6	72.4										
Tetracycline	57				39.7	3.4			1.7	55.2			
Trimethoprim-Sulfametoxazol	0				100								

^1^Unshaded cells indicate the range of concentrations tested for each antimicrobial agent. Shaded cells indicate concentrations outside the range tested for each substance. MIC equal to or lower than the lowest concentration tested for an antibiotic substance (≤Y μg/mL), is given as a percentage at the lowest tested concentration. Blank unshaded cells indicate lack of isolates with that MIC. Bold vertical lines indicate epidemiological cut-off values retrieved from the European Committee on Antimicrobial Susceptibility Testing or, for cephalotin and trimethropim-sulfametoxazole, clinical breakpoints from SVA.

Only six isolates from three herds (A, B and D) were lactose-positive based on phenotyping, PCR, and genomic analysis, with full agreement between methods. Lactose fermentation was observed at the first visual assessment after 24 hours of incubation. Five lactose fermenting isolates originated from milk, and one from the nasal mucosa of a calf. Four lactose fermenting isolates belonged to ST616, and one each to ST617 and ST1652. There was an association between lactose fermentation and ST, with ST616 being overrepresented among lactose fermenting strains (4 out of 6) (*p* = 0.05), but no association between lactose operon variant (Lac.2b, n = 1; Lac2.d, n = 5) and sample type.

## Discussion

### Nasal carriage of GBS is highly prevalent in camels

We found a high prevalence of GBS on the nasal mucosa of apparently healthy animals (27%), similar to the findings by Younan and Bornstein [13], but in contrast to the low prevalence described in a different study in northern Kenya [38]. Furthermore, isolates from the nasal mucosa showed a high level of genetic diversity, with isolates belonging to all STs identified in the study, with the exception of ST616, an ST which is significantly associated with the mammary gland [14, 39]. These findings suggest that nasal colonisation of GBS in healthy camels is common, in contrast to the situation in humans and cattle. In humans, prevalence of nasopharyngeal carriage is estimated at around 10% in different geographic regions [28, 40, 41]. The observation of a camel-calf pair with a shared nasal GBS type suggest nose to nose contact may be a route of transmission. Reports of GBS in respiratory tract infections in camels suggest that the nasal cavity may serve as a reservoir for opportunistic GBS infections [13, 42].

In humans, rectal colonisation is the most common form of GBS carriage and widely recognized as a source of sporadic disease in infants [43]. By contrast, we observed a very low prevalence (3%) from rectal swabs in camels, similar to a previous report where none of 25 camel rectal samples tested positive for GBS [13], and to the situation in cattle [10, 11]. The finding of two genetically closely related isolates, originating from a rectal sample from a suckling calf and milk from its mother, suggests the probable passage of GBS through the gastrointestinal tract in camels. It is, however, not possible to say whether these findings are the results of ingestion of GBS infected milk by the calf or if it is indicative of rectal colonisation. Vaginal colonisation was not observed in our study, suggesting absence or a low prevalence, compatible with a reported prevalence of vaginal GBS carriage in camels of 1 to 21% [13]. Vaginal colonisation is relatively rare in cattle [11]. By contrast, and mirroring the prevalence of rectal colonisation, vaginal prevalence of GBS in humans is much higher, generally estimated to be in the range of 20 to 30% [43]. Thus, our data imply that the niche-predilection of GBS differs between host species, with preferred sites being the rectum and urogenital tract in humans, the mammary gland in cattle, and the nasal mucosa in camels. For *Staphylococcus aureus*, another important mastitis pathogen of ruminants and camelids, similar differences in niche-predilection have been described, with the mammary gland being the most common site of *S*. *aureus* isolation in cattle, and the nose the most common site in sheep [44].

Another striking difference between camel isolates and those from humans and cattle is the distribution of serotypes. Serotype VI represented 53% of isolates typed and was the most common serotype in the current study, but this serotype is very rare among human GBS, to the point where it is not included in current human GBS vaccine trials [45]. Likewise, in dairy cattle, serotype VI is exceedingly rare, with a prevalence of <1% among 171 isolates in two global studies of bovine GBS [46, 47]. Serotype IV was the second most common type in camels in our study. This is an emerging serotype in humans, i.e., historically of limited relevance but increasingly recognised as a cause of disease [48]. Some STs with serotype IV are found in humans as well as cattle, notably GBS ST196 [49], but there is no evidence for occurrence of any of the camel-associated STs with serotype IV in humans. Strikingly, serotypes Ia, Ib and V, which are common in human and bovine GBS [46, 47, 49] were absent from the camel GBS population in our study. Fischer and colleagues [14] also describe a relatively high prevalence (26%) of serotype VI among camel GBS isolates and absence of serotype Ib. Their results differ from ours in that they detected serotypes Ia (37%), III (27%) and, occasionally, II and V, whereas they do not report detection of serotype IV.

### Extramammary GBS strains are a potential source of mastitis in camels

Only four of ten GBS milk isolates belonged to ST616, which has previously been associated with mastitis [14, 39]. The remaining milk isolates belonged to STs that were predominantly found in extramammary sources. Thus, GBS mastitis in camels may have a mixed epidemiology, comprising transmission from udder to udder (“contagious transmission”) and transmission from extramammary sources (“environmental transmission”). Although GBS was isolated from rectal samples on a few occasions in this study, this is not a likely route for within-herd transmission in camel herds, considering that camels are mostly kept in an arid environment and that camel faeces are unlikely to contaminate the environment due to their dry and firm consistence, in contrast to the situation for high-producing or housed dairy cattle. Transmission from skin or mucosa, as described for *S*. *aureus* in cattle [50, 51], seems more likely considering our findings. The existence of extramammary GBS on the nasal and oral mucosa of calves also points towards calves acting as reservoirs for GBS, which could contribute to transmission of extramammary isolates to the udder, as shown for *S*. *aureus* in sheep [44]. In contrast to what has previously been reported [52], we observed cross-suckling on a few occasions. It has been suggested that suckling calves can cause contamination of the camel udder [53] and contribute to udder-to-udder transmission of GBS.

We should also consider the possibility of false-positive results from milk cultures because any detection of GBS from milk was considered indicative of IMI in our study, as is customary in specialized mastitis diagnostic laboratories [54]. The rationale behind this approach in dairy cattle is the perception that GBS is an obligate intramammary pathogen, combined with concern about the possibility of contagious transmission and GBS outbreaks within herds. Both for dairy cattle [10–12] and for camels [14; this study], the notion of an obligate intramammary pathogen has been disproven. Even so, we consider GBS detection in our milk samples to constitute evidence of infection because samples were collected aseptically by a trained professional, and only from camels with evidence of mastitis, which increases the a priori prevalence of GBS and hence the positive predictive value of the culture results.

Herds E and F had comparatively high isolation rates of GBS from the nasal and oral mucosa of calves but no GBS was found in milk. This observation may result from our study design, whereby not all camels’ udders were sampled, but only those with CMT ≥ 2 in at least one quarter. GBS has previously been isolated from quarters without an elevated cell count [7] and similarly, from dairy cows with a composite somatic cell count of <200,000 cells/mL [55]. Hence, it is possible that camels with GBS mastitis could have passed undetected in this study. It may explain how ST616 could be detected on the oral mucosa of a calf without having been detected in the milk of its mother (pair F3, **Fig 3**). This limitation in animals selected for milk sampling was unfortunately necessary to ensure animal owner compliance with the sampling procedure. However, the presence of GBS located exclusively in extramammary sites in these two camel herds, cannot entirely be ruled out.

The association between specific GBS types and intramammary infection was thought to be due to the presence of the lactose operon (Lac.2), both in cattle [25, 47, 49] and in camels [39]. In a study on camels from a different region in Kenya and with a different production system, more than 80% of GBS isolates from milk harboured Lac.2 [39]. In the current study, Lac.2 was only detected in 40% of milk isolates. The ability to ferment lactose likely offers GBS an evolutionary advantage in adaption to the mammary gland, however, it appears that for camel GBS this is not a necessary feature to establish intramammary infections. As this study was cross-sectional, we cannot determine whether the presence of a Lac.2 operon was associated with chronicity of infection.

### The local context affects the epidemiology of GBS in camel herds

The phylogenetic analysis revealed distinct herd clusters, reflecting how ranches are relatively closed management systems in comparison to traditional pastoral herds. This outcome is quite distinct from previous results for milk isolates from traditional pastoralist herds, where highly similar strains were identified in multiple herds [39]. Camel herdsmen working on ranches visit each other and ranch camel herds and other local sedentary camel herds in Laikipia are not strictly separated from each other. This may account for the between-herd transmission, as illustrated by the findings of genetically related isolates in herd A, E and F. Additional known epidemiological risk factors for between-herd transmission, would be close neighbours having frequent contact, and household members from herd F being employed at the ranch of herd A. Considering the role of local context in GBS epidemiology, our study provides proof-of-principle for the existence of extramammary GBS in camels, in agreement with results from Fisher and colleagues [14], and on potential routes of GBS transmission, but our findings cannot necessarily be extrapolated to all camel populations in the Horn of Africa or beyond.

Prevalence of tetracycline non-WT among GBS isolates was herd-associated and ST-associated, with non-WT being present in all isolates from two herds compared to none or almost none from two other herds. The *tet*(M) gene, one of the most common genes for tetracycline resistance, was found in all non-WT isolates. This gene, which is carried by integrative conjugative elements, has been described in GBS in camels as well as other host species, notably humans [14, 56]. In four isolates, we detected the *tet*(L) gene which has not previously been reported in camel GBS. The observed differences in susceptibility patterns among isolates originating from different herds herds and STs are likely due to the combination of selective pressure from antimicrobial use, horizontal gene transfer of resistance genes between GBS lineages, and transmission of GBS lineages with or without resistance genes within and between herds. Antibiotic sales and use in Kenya are largely unmonitored [57], but antimicrobials, particularly tetracycline, are reportedly a common treatment option against infections in camels [58, 59]. Interestingly, isolates originating from milk were more likely to be tetracycline resistant than isolates from other sampling sites. This is in agreement with previous findings of high levels of tetracycline resistance in GBS from milk [7, 53] and tetracycline resistance being less common in GBS from the nasal mucosa in camels [38]. In cattle, treatment of mastitis with tetracycline has been shown to be inefficient and results in subtherapeutic concentrations in the udder, something that would promote development or acquisition of resistance in udder pathogens [60, 61]. For example, exposure to tetracycline and other subtherapeutic concentrations of ribosome-targeting antibiotics, e.g., macrolides, lincosamides, and streptogramins may induce transfer of Tn*916* [62], a mobile genetic element that facilitates horizontal transfer of resistance in GBS from humans and camels [14, 56].

## Conclusions

In conclusion, the prevalence of GBS in the nares of camels in our study population was higher than observed in any other GBS host species. In addition, occasional oral carriage and faecal shedding were detected but no vaginal carriage, in stark contrast to the situation in humans, were rectal and vaginal carriage are far more common than oropharyngeal colonisation, or the situation in cattle, where all extramammary detection of GBS is relatively rare. The serotype distribution of GBS was markedly different between camels and its other major mammalian hosts (humans and cattle), adding further evidence that the ecology of GBS in camels is unique. Of five STs that were detected, only ST616 was significantly associated with milk and was the only ST not found in the nares. However, the majority of isolates from milk belonged to STs that were also detected in extramammary sources, suggesting that they may play an important role in the epidemiology of mastitis in camels, which is in contrast to the situation in cattle.

## Supporting information

S1 Dataset Authors’ original data for 57 GBS-isolates collected from camels in Laikipia County, Kenya, October—November, 2019. The dataset includes accession numbers available at European Nucleotide Archive (ENA), information regarding herd identity, source of the isolate, sequence type (ST), capsular serotype, minimum inhibitory concentration (MIC) values for phenotypic antimicrobial resistance testing, presence/absence of antimicrobial resistance (AMR) genes, phenotypic lactose fermentation results, Lac.2 PCR results, presence/absence of the Lac.2 operon genotypes and herd-coordinates.
(XLSX)
